# Apnea–Hypopnea Index Versus Hypoxic Burden as Predictors of Blood Pressure Response to Continuous Positive Airway Pressure Treatment in Patients With Obstructive Sleep Apnoe

**DOI:** 10.1111/jch.70262

**Published:** 2026-05-22

**Authors:** Sebastian Bertram, Diana Racovitan, Adrian Doevelaar, Felix S. Seibert, Nina Babel, Simon Wang, Timm H. Westhoff, Maximilian Seidel

**Affiliations:** ^1^ Medical Department 1 University Hospital Marien Hospital Herne, Ruhr‐University Bochum Herne Germany; ^2^ Center For Translational Medicine University Hospital Marien Hospital Herne, Ruhr‐University Bochum Herne Germany

**Keywords:** blood pressure, continuous positive airway pressure, hypertension, hypoxic burden, sleep apnoe

## Abstract

Obstructive sleep apnea (OSA) is associated with increased cardiovascular risk and hypertension. While continuous positive airway pressure (CPAP) therapy reduces blood pressure (BP), its effect shows substantial interindividual variability. Hypoxic burden (HB) has been proposed as a potentially superior predictor of cardiovascular outcomes compared to the apnea–hypopnea index (AHI).

In this retrospective study, 141 patients with OSA underwent in‐laboratory polysomnography and initiated CPAP therapy. Office BP and clinical parameters were assessed at baseline and follow‐up (median 200 days). Receiver operating characteristic (ROC), correlation, multiple linear and logistic regression analyses were performed to compare the predictive value of HB and AHI for BP reduction.

CPAP therapy resulted in a modest, non‐significant reduction in systolic BP(−2.3 mmHg ±17.7,0.1237), while diastolic BP remained unchanged. Higher AHI and HB levels were associated with higher baseline systolic BP and numerically greater BP reductions, particularly in individuals with a higher than the median HB ≥57.5%min/h or median AHI ≥34.3/h. However, neither HB nor AHI predicted a systolic BP reduction ≥5 mmHg (AUC values≈0.5), and no relevant correlations between AHI, HB and blood pressure response were observed.

HB is associated with OSA severity and higher baseline BP. However, it is not superior to AHI in the prediction of a CPAP‐associated reduction of BP in patients with OSA.

## 1 Introduction

Obstructive sleep apnea (OSA) is associated with increased cardiovascular risk including hypertension [1, 2, 3, 4]. OSA contributes to arterial hypertension in various ways. The main causes are sympathetic activation, activation of the renin‐angiotensin‐aldosterone system, fragmented sleep, inflammation and vascular remodeling [5, 6, 7, 8]. Continuous positive airway pressure (CPAP) therapy leads to modest reductions in blood pressure, with meta‐analyses reporting mean decreases of approximately 3 mmHg systolic and 2 mmHg diastolic [9]. This effect occurs even in resistant hypertension and increases with baseline blood pressure, young age, and the severity of oxygen desaturations [10, 11]. The effects of CPAP exceed the blood pressure lowering effects of nocturnal oxygen supplementation [12]. The CPAP‐related reduction in blood pressure could not be predicted by AHI [13].

Data from observational studies suggest that CPAP therapy could provide cardiovascular protection [14, 15]. To date, no randomized study (SAVE, ISAACC, RICCADSA) with a cumulative total of 3549 patients has been able to demonstrate a CPAP‐induced effect on the reduction of cardiovascular events in patients with pre‐existing cardiovascular disease [16, 17, 18]. These findings and the moderate blood pressure improvement could suggest that clinical use of CPAP might primarily focus on improving symptoms and quality of life. Evidence from community cohorts points to two central markers of OSA severity and prediction of cardiovascular events: event‐related heart rate acceleration and the overall burden of hypoxemia ('hypoxic burden) [19, 20, 21]. A recently published post hoc analysis of these studies identified a subgroup classified as high‐risk OSA (heart rate response >9.4beats/min or a hypoxic burden (HB) of >87.1% min/h) that benefited from CPAP [22]. Based on a prior study showing that more severe desaturation is linked to a greater blood pressure response to CPAP therapy, evaluating hypoxic burden as a potential predictor is clinically important [11]. Moreover, reductions in hypoxic burden have been associated with clinically relevant decreases in systolic blood pressure, although prior studies were limited by confounding factors such as concomitant oxygen therapy [23]. In summary, a more precise phenotyping of OSAS patients is crucial to improve predictions of cardiovascular risk reduction and to identify those who are most likely to benefit from CPAP therapy, particularly with regard to cardiovascular outcomes and individualized blood pressure lowering. We therefore formulated the hypothesis that hypoxic burden, would be more suitable for predicting a reduction in blood pressure, and compared this with the AHI. From a clinical perspective, it would be of great interest to define thresholds for HB and AHI that are associated with a high probability of a clinically relevant reduction in blood pressure. Ettehad et al. reported that each 1 mmHg reduction in systolic blood pressure is associated with an approximately 2% relative reduction in the risk of major cardiovascular events, suggesting that a 5 mmHg reduction may translate into an estimated 10% relative risk reduction [24]. The present study therefore evaluates the association of HB and AHI with CPAP‐induced changes in systolic blood pressure and explores whether specific thresholds of HB and AHI are associated with a clinically meaningful reduction in systolic blood pressure (≥5 mmHg). This retrospective analysis is the first to examine prediction of blood pressure reduction by HB using polysomnography.

## 2 Methods

### 2.1 Patients and Protocol

We performed a retrospective study of polysomnographic recordings and clinical data of patients undergoing in‐laboratory polysomnography in a German university hospital. We made use of an electronic data extraction approach to identify subjects suffering from obstructive sleep apnea who had been admitted from December 2023 to September 2025.

**FIGURE 1:**
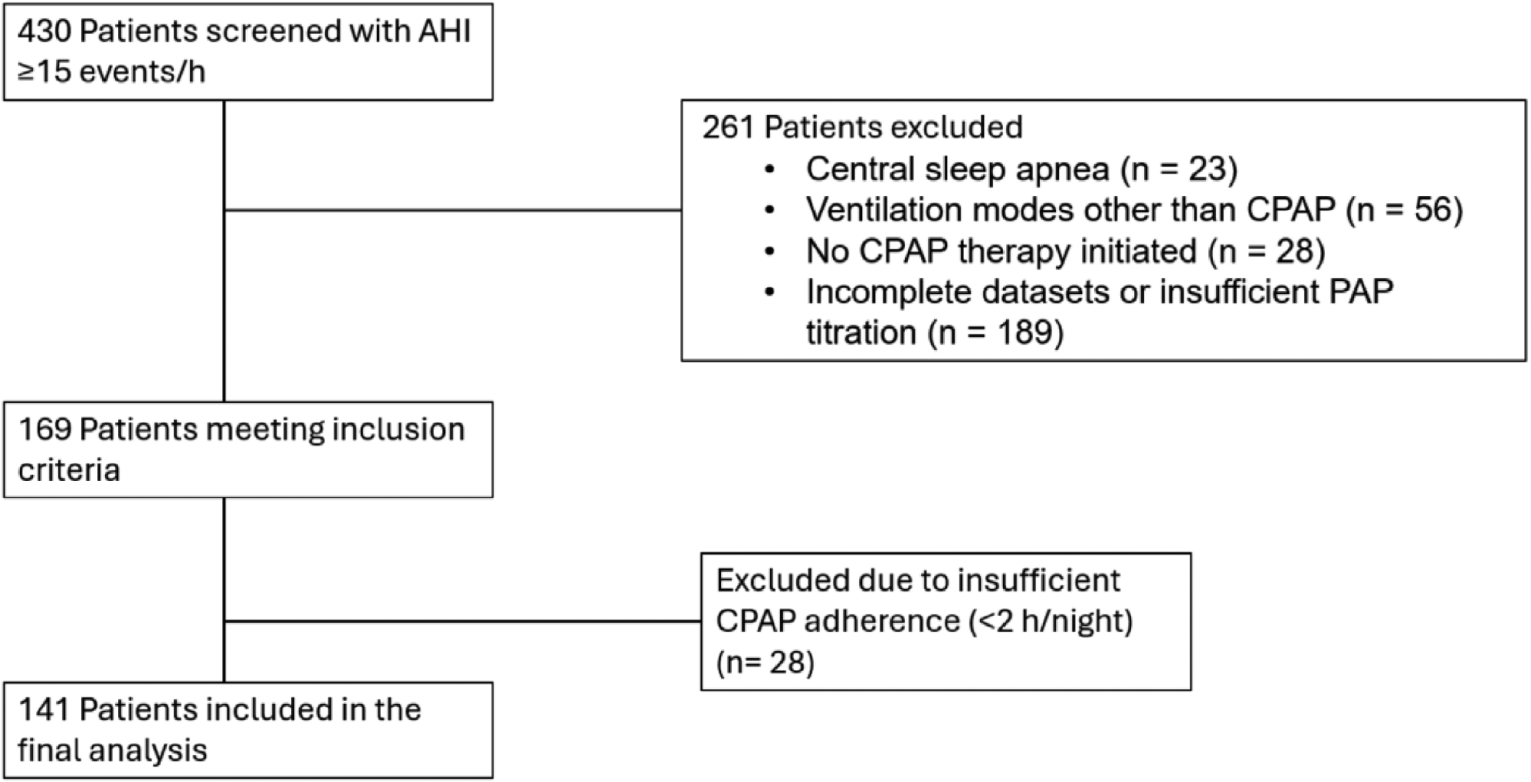
Flow diagram of patient selection. Of 430 patients screened (AHI ≥15 events/h), 261 were excluded (central sleep apnea, non‐CPAP ventilation modes, no CPAP therapy, or incomplete datasets). A total of 169 patients met inclusion criteria, of whom 28 were further excluded due to insufficient CPAP adherence (<2 h/night), resulting in 141 patients included in the final analysis.

Inclusion criteria were a diagnosis of obstructive sleep apnea indicated by an apnea‐hypopnea index (AHI) ≥15/h, initiation of positive airway pressure (PAP) therapy and an age of at least 18 years. Exclusion criteria were pre‐established PAP therapy, central sleep apnea, ventilation modes other than CPAP, changes in antihypertensive medication, missing BP measurements at baseline and/or follow‐up, and an average PAP therapy usage time of <2 h per night. Clinical data were collected during polysomnography at the start of the study and during the first follow‐up examination after PAP titration. 430 patients with an AHI ≥15 events/h were screened. After exclusion of 261 patients due to central sleep apnea, use of ventilation modes other than CPAP, no initiation of CPAP therapy or insufficient PAP titration and incomplete datasets, 169 patients met the inclusion criteria. Of these, 28 patients were excluded due to insufficient CPAP adherence (<2 h/night), resulting in a final study population of 141 (Figure 1) patients included in the analysis. Follow‐up polysomnography was conducted after a median of 200 days following PAP titration. Daytime sleepiness was assessed using the Epworth Sleepiness Scale (ESS) questionnaire. BP was assessed at baseline and follow‐up.

Polysomnographic monitoring comprised simultaneous noninvasive recordings of electroencephalogram, electrooculogram, submental and bilateral leg electromyogram, nasal or oral airflow, oxygen saturation (pulse oximetry), snoring microphone, chest and abdominal respiratory movement, body position, and electrocardiogram. Polysomnography results were scored according to standard criteria of the American Academy of Sleep Medicine guidelines by an experienced observer [25]. The study was conducted in accordance with the declaration of Helsinki and was approved by the ethics committee of Ruhr‐University Bochum (reference number 18‐6725‐BR). The need for patient approval and informed consent was waived by ethics committee of Ruhr‐University Bochum due to the retrospective nature of the study.

### 2.2 Blood Pressure Measurement

Office blood pressure was assessed auscultatory according to Riva‐Rocci at admission by trained observers in a seated position in a quiet room of the laboratory after a resting time of ≥ 5 min. In a standardized setting, a single measurement was taken at each physical examination. Measurements were performed at daytime during a physical examination prior to the polysomnography. The cuff size was chosen depending on the upper arm circumference using a standard bladder cuff 13×24 cm for a circumference of 24–32 cm, 15×30 cm for a circumference of 33–41 cm, and 12×22 cm for a circumference of <22 cm. The cuff was deflated by a maximum of 3 mmHg per second.

### 2.3 Statistical Analysis

Data were checked for normality of distribution by the D`Agostino & Pearson test. Normally distributed data were presented as mean ± standard deviation and were compared from baseline to follow up by a paired two‐tailed *t*‐test. Categorical data and nonnormally distributed numeric data are presented as median and interquartile range (IQR).

Correlation analyses between AHI, HB were performed according to Spearman. To assess independent associations with systolic blood pressure change, a multivariable linear regression analysis was performed including AHI, HB, ΔHB/ΔAHI and adjusting for potential confounders. Receiver‐operating characteristic (ROC) curve analyses were performed in an attempt to predict a SBP reduction at least 5 mmHg and at least 10 mmHg by baseline AHI and HB. Multiple logistic regression analyses were performed in an attempt to predict a SBP reduction of at least 5 mmHg by baseline AHI and HB. As sensitivity analyses, responder thresholds for systolic blood pressure reduction (≥5 mmHg and ≥10 mmHg) were evaluated. Participants were stratified according to predefined cutoffs of hypoxic burden (HB) and apnea–hypopnea index (AHI), and the proportion of individuals achieving these reductions was calculated with corresponding 95% confidence intervals.

To assess the incremental predictive value of sleep apnea metrics, a prespecified multivariable logistic regression base model including relevant clinical covariates was constructed. Model discrimination and fit were evaluated after adding either the apnea–hypopnea index (AHI) or hypoxic burden (HB) to the base model using the area under the receiver operating characteristic curve (AUC) with 95% confidence intervals and the Akaike information criterion (AIC). *P* < 0.05 was regarded statistically significant. All statistical analyses were performed using GraphPad Prism (Version 10.6.1, GraphPad Software, La Jolla California, USA)

### 2.4 Sample Size Estimation

With regard to studies on the effect of CPAP on blood pressure in subjects with obstructive sleep apnea, we anticipated an effect of 3 mmHg on systolic blood pressure with a standard deviation of 10 mmHg for the sample size calculation. Based on a paired two‐tailed *t* test, a study size of 90 is necessary to achieve a power of 80% and a two‐sided level of significance of 5%, for detecting this difference in blood pressure. We aimed to exceed this number by > 20% for drop‐outs due to incomplete study data.

## 3 Results

After screening 169 data sets, 141 fulfilled all in‐ and exclusion criteria and were included in the analysis. The study population had a median age of 62 (IQR 55–71). 48 patients (33.1%) of the study population were female. 93 patients (66%) had hypertension. The median number of antihypertensive drugs in the overall study population was 1 (IQR 0–2) at baseline and follow‐up. The most prevalent further cardiovascular comorbidities were diabetes (*n* = 33, 23.5%), coronary heart disease (*n* = 24, 17%) and hyperlipidemia (*n* = 54, 61.7%). Baseline AHI was 33.3 (24.3–52.8) per hours. The baseline HB was 57.7 (38.1–136) %min/h. Baseline median ESS index was 8 (IQR 5–12). The average PAP usage per day was 5.5 (3.7–7.1) h. The duration between PAP titration and follow up was 200 (186–229) days. Epidemiological and clinical baseline parameters are presented in Table 1.

**TABLE 1:** Epidemiological and clinical baseline parameters of the study population.

**Study population (*n* = 145)**	
Age (years)	62 (55–71)
Body mass index (kg/m^2^)	32.5 (28.9–36.9)
Gender Female Male	48 (33.1%) 97 (66.9%)
Apnea hypopnea index (AHI) (events/h)	33.3 (24.3–52.8)
AHI change (events/h)	29.3(20.2–45)
Hypoxic burden (%min)/h	57.7 (38.1–136)
Hypoxic burden change (%min)/h	52.5 (33–126)
Epworth sleepiness scale score	8 (5–12)
Number of antihypertensive drugs baseline	1 (0–2)
Number of antihypertensive drugs follow up	1 (0–2)
Average PAP usage per day (h)	5.5 (3.7–7.1)
Days between titration and follow up	200 (186–229)
**Cardiovascular disease**	
Arterial fibrillation	8 (5.7%)
Coronary heart disease	24 (17%)
**Cardiovascular risk factors**	
Hypertension	93 (66%)
Smoking	45 (31.9%)
Hyperlipidemia	54 (61.7%)
Diabetes	33 (23.4%)
**Laboratory findings**	
Creatinine (mg/dl)	1 (0.8–1.1)
Estimated glomerular filtration rate (eGFR) (ml/min/1.73m^2^)	79–9 +−20.3
Low‐density lipoprotein (LDL) cholesterol (mg/dl)	115 (78–147.5)
High density lipoprotein (HDL) cholesterol (mg/dl)	49 (41–60.5)
C‐reactive protein (CRP) (mg/dl)	0.2 (0.1–0.5)
Uric acid (mg/dl)	6 (5.1–7)
HbA1c (Glycated hemoglobin A1c) (%)	5.9 (5.6–6.5)

**FIGURE 2:**
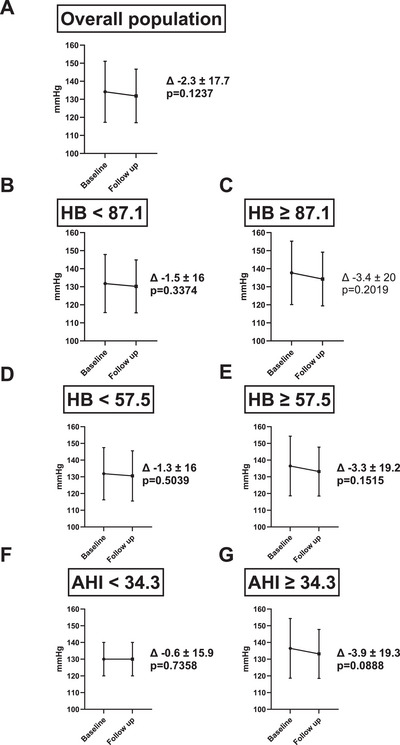
Systolic blood pressure before and 200 (186–229) days after initiation of continuous positive airway pressure therapy in *n* = 141 patients with sleep apnea. Cut‐off values for AHI (≥34.3) and HB (≥57.5) were defined using the median values obtained from polysomnography. Additionally, the high‐risk OSA cut‐off for HB (≥87.1) was based on a previously published definition. Plots provide mean and standard deviation in (A) the overall study population, (B–C) stratified by definition of high and low risk OSA (HB ≥87.1%min/h, HB <87.1% min/h), (D–E) stratified by HB higher or lower than median and (F–G) by apnea–hypopnea index (AHI) higher or lower than median. Data are provided as mean and standard deviation. The change in systolic blood pressure from baseline to follow‐up (ΔSBP), including corresponding *p*‐values for within‐group comparisons, is displayed in the figure. *P*  <  0.05 was considered significant.

Baseline systolic blood pressure (SBP) for the general population was 134.2 ± 16 mmHg. Diastolic blood pressure (DPB) was 81.1 ±11.04 mmHg. SBP and DBP decreased from baseline to follow up (SBP 131.9 ± 14.83 mmHg, *p* = 0.1237, DPB 80.5±10.34 mmHg, *p* = 0.6784). The change of SBP and DBP is summarized in Table 2 and Figure 2.

**TABLE 2:** Mean systolic and diastolic blood pressure before and 200 (186–229) days after initiation of continuous positive airway pressure therapy in 141 patients with sleep apnea. Mean changes (Δ) in blood pressure from baseline to follow‐up were analyzed in relation to AHI and hypoxic burden (HB), using median‐derived cut‐offs for AHI (≥34.3) and HB (≥57.5) as well as the previously defined high‐risk HB threshold for obstructive sleep apnea (≥87.1).

	Blood pressure baseline	Blood pressure follow up	Δ Mean blood pressure change	*p*
**Overall population *n* = 141**				
Systolic	134.2 ± 16.9	131.9 ± 14.8	−2.3 ± 17.7	0.1237
Diastolic	81.1 ±11	80.7±10.3	−0.4 ± 12.8	0.6784
**HB ≥57.5 *n* = 71**				
Systolic	136.5 ± 17.9	133.2 ± 14.6	−3.3 ± 19.2	0.1515
Diastolic	80.6± 12	80.3 ± 10.4	−0.4 ± 12.5	0.8125
**HB <57.5 *n* = 70**				
Systolic	131.9 ± 15.6	130.6 ± 15	−1.3 ± 16	0.5039
Diastolic	81.6 ± 10	81 ± 10.4	−0.5 ± 13.16	0.7311
**HB ≥87.1 *n* = 57**				
Systolic	137.7 ± 17.6	134.3 ± 14.9	−3.4 ± 20	0.2019
Diastolic	80.8 ± 11.7	81.1 ± 10.3	0.3 ± 12.44	0.8737
**HB <87.1 *n* = 84**				
Systolic	131.8 ± 16.1	130.2 ± 14.6	−1.5 ± 16	0.3774
Diastolic	81.3 ± 10.6	80.4 ± 10.5	−0.9 ± 13	0.5156
**AHI ≥34.3 *n* = 71**				
Systolic	136.8 ± 17.5	132.9 ± 15.4	−3.9 ± 19.3	0.0888
Diastolic	81.2 ± 11.4	81.2 ± 10.5	0.03 ± 13.1	0.9856
**AHI <34.3 *n* = 70**				
Systolic	131.5 ± 16	130.9 ±14.3	−0,6 ± 15.9	0.7358
Diastolic	81 ± 10.8	80.1±10.2	−0.9 ± 12.5	0.5350

To test the hypothesis that the number of apnea events or the HB predict the blood pressure (BP) response to PAP therapy, we compared individuals with an AHI/HB greater than the median with subjects below the median. According to recently published data on predicting the cardiovascular benefits of PAP therapy in individuals reaching an HB of ≥87.7 %min/h, we additionally compared individuals with an HB ≥87.7 %min/h with individuals with lower HB levels.

As presented in Table 2, patients with an AHI ≥34.3/h showed a numerically greater reduction in systolic BP from baseline to the first follow‐up compared with patients with an AHI <34.3/h. The systolic BP change was −3.9 ± 19.3 mmHg in the AHI ≥34.3/h group and −0.6 ± 15.9 mmHg in the AHI <34.3/h group. When dividing the cohort by HB levels (HB ≥57.5 %min/h vs. HB <57.5 %min/h), the systolic BP change was −3.3 ± 19.2 mmHg versus −1.3 ± 16.0 mmHg. The change in diastolic BP was numerically greater in the groups with an AHI or HB below the respective median values, with −0.5 ± 13.16 mmHg for HB <57.5 %min/h and −0.9 mmHg for AHI <34.3/h. A small numerical increase appeared in the AHI ≥34.3/h group (DBP change: 0.03 ± 13.1 mmHg). In the HB ≥57.5 %min/h group, the diastolic BP change was −0.4 ± 12.5 mmHg.

**FIGURE 3:**
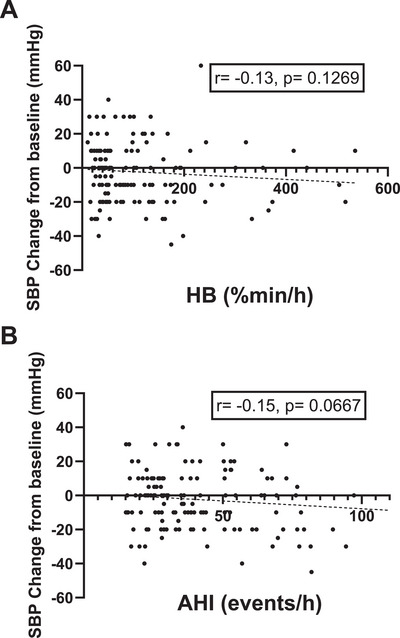
Correlation of (A) hypoxic burden (HB) and (B) apnea–hypopnea index (AHI) with systolic blood pressure changes at follow‐up after CPAP initiation. No association between HB or AHI and systolic blood pressure changes was observed.

Spearman correlation showed no association between SBP response and AHI (*r* = 0.11, *p* = 0.2065) or HB (*r* = 0.09, *p* = 0.2807). (Figure 3, Table S1)

In multivariable linear regression analysis, baseline systolic blood pressure (*β* = −0.73, 95% CI −0.88 to −0.58, *p* < 0.0001) and BMI (*β* = 0.55, 95% CI 0.16 to 0.94, *p* = 0.0063) were independently associated with changes in systolic blood pressure, whereas neither AHI (*β* = −0.04, 95% CI −0.21 to 0.13, *p* = 0.6395) nor HB (*β* = 0.01, 95% CI −0.03 to 0.04, *p* = 0.7158) showed a significant association with blood pressure change after adjustment for clinical covariates. (Table 3) In the adjusted multivariable linear regression model, neither ΔAHI nor ΔHB was independently associated with ΔSBP, whereas baseline systolic blood pressure remained a significant predictor of BP change. (Table S2) Consistently, in multivariable logistic regression analysis evaluating predictors of systolic blood pressure response, higher baseline systolic blood pressure was independently associated with BP response (OR 1.10, 95% CI 1.06–1.14, *p* < 0.0001), while neither AHI (OR 1.02, 95% CI 0.98–1.05, *p* = 0.3554) nor HB (OR 0.99, 95% CI 0.99–1.00, *p* = 0.5870) were associated with BP response. (Table 4)

**TABLE 3:** Multivariable linear regression analysis of predictors of change in systolic blood pressure after CPAP therapy. The model included AHI, hypoxic burden (HB), baseline systolic blood pressure, antihypertensive treatment intensity (>3 vs. ≤3 drugs), CPAP adherence (h/night), follow‐up time, BMI, age, sex, and comorbidities. Results are presented as *β*‐coefficients with 95% confidence intervals (CI). Baseline systolic blood pressure and BMI were significantly associated with BP change, whereas AHI and HB were not independent predictors. *n* = 141; adjusted *R* ^2^ = 0.40.

Variable	*ß*‐coefficient	95%CI	*p* value
Apnea–hypopnea index (AHI)	−0.04	−0.21 to 0.13	0.6395
Hypoxic burden (HB)	0.01	−0.03 to 0.04	0.7158
Baseline systolic BP(mmHg)	−0.73	−0.88 to −0.58	<0,0001
Antihypertensive regimen (>3 vs. ≤3 drugs)	−2.6	−11 to 6.1	0.5529
CPAP usage (h/night)	0.61	−0.45 to 1.7	0.2605
Follow up time (days)	0.01	−0.03 to 0.05	0.6714
Body mass index (kg/m^2^)	0.55	0.16 to 0.94	0.0063
Age (years)	0.13	−0.07 to 0.33	0.2071
Sex (male vs. female)	−4.6	−9.9 to 0.78	0.0935
Arterial hypertension	1.3	−4.0 to 6.6	0.6300
Diabetes	−3.2	−9.3 to 2.8	0.2866
Coronary heart disease	−0.49	−7.4 to 6.4	0.8877
Hyperlipidemia	2	−3.2 to 7.2	0.4404

**TABLE 4:** Multivariable logistic regression analysis of predictors of systolic blood pressure response after CPAP therapy. Multivariable logistic regression was performed to identify independent predictors of systolic blood pressure response (≥5 mmHg reduction). The model included AHI, hypoxic burden (HB), baseline systolic blood pressure, antihypertensive treatment intensity (>3 vs. ≤3 drugs), CPAP adherence, follow‐up time, BMI, age, sex, and comorbidities. Results are presented as odds ratios (OR) with 95% confidence intervals (CI). *n* = 141. BP response was defined as systolic blood pressure reduction ≥5 mmHg between baseline and follow‐up. Continuous variables are presented per unit increase. *n* = 141; AUC = 0.81 (95% CI 0.74–0.88), *p* < 0.0001; Tjur's *R* ^2^ = 0.30; Hosmer–Lemeshow *p* = 0.46.

Variable	Odds ratio (OR)	95%CI	*p*
Apnea–hypopnea index (AHI)	1.02	0.98–1.05	0.3554
Hypoxic burden (HB)	0.099	0.99–1.00	0.5870
Baseline systolic BP(mmHg)	1.10	1.06–1.14	<0.0001
Antihypertensive regimen (>3 vs. ≤3 drugs)	3.26	0.68–17.6	0.1419
CPAP usage (h/night)	1.04	0.86–1.26	0.7074
Follow up time (days)	1.00	0.99–1.01	0.9384
Body mass index (kg/m^2^)	0.94	0.87–1.00	0.0609
Age (years)	0.98	0.95–1.02	0.3844
Sex (male vs. female)	2.17	0.86–5.73	0.1037
Arterial hypertension	1.08	0.44–2.66	0.8669
Diabetes	1.75	0.58–5.36	0.3208
Coronary heart disease	0.63	0.16–2.24	0.4779
Hyperlipidemia	0.85	0.34–2.13	0.7265

**FIGURE 4:**
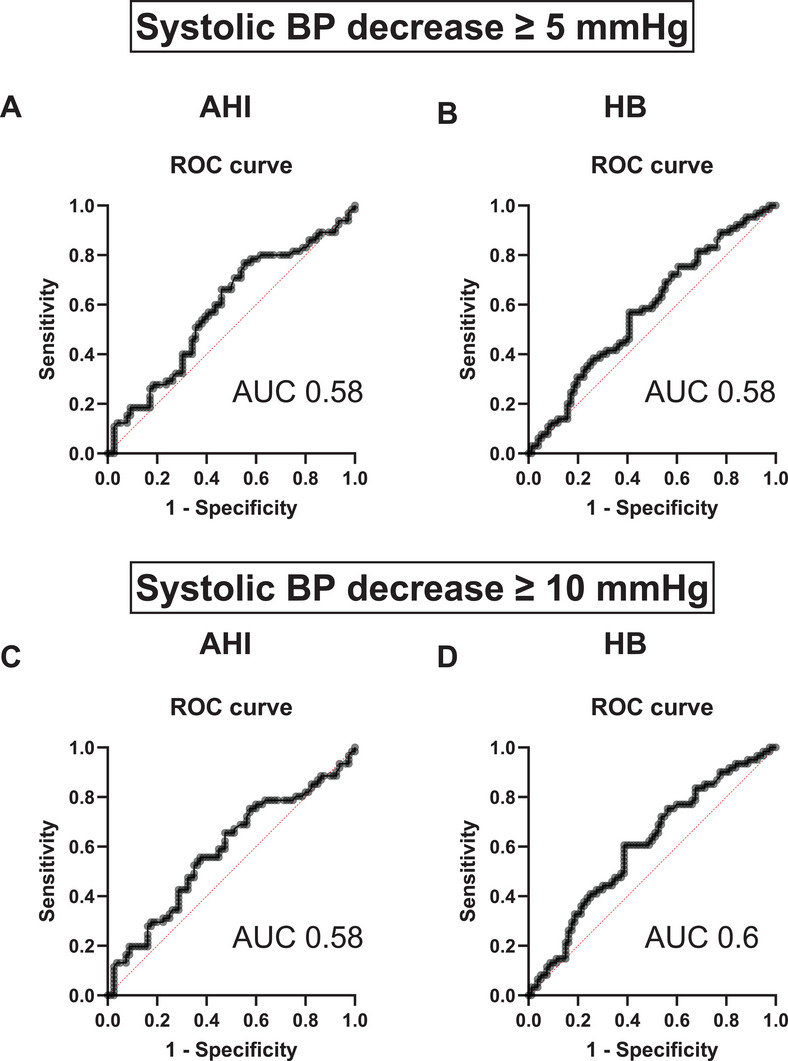
Receiver‐operating characteristics analysis for a continuous positive airway pressure‐induced SBP difference at least 5 mmHg and at least 10 mmHg in dependence of apnea–hypopnea index (AHI) and hypoxic burden (HB).

In an attempt to define an AHI or HB cut‐off value for predicting an SBP decrease of at least 5 or 10 mmHg, ROC analyses were performed. The areas under the curve (AUC) for an SBP decrease of 10 mmHg were 0.58 (AHI) and 0.60 (HB). Accordingly, the AUC values for an SBP decrease of at least 5 mmHg were 0.58 for both AHI and HB. (Figure 4)

As sensitivity analyses, responder thresholds for SBP reduction were evaluated. SBP reduction ≥5 mmHg occurred in 52.1% (95% CI 40.7–63.3) of participants with HB ≥57.5 compared with 40.0% (95% CI 29.3–51.7) in those with HB <57.5; corresponding proportions for SBP reduction ≥10 mmHg were 52.1% (95% CI 40.7–63.3) and 34.3% (95% CI 24.2–45.9), respectively. When applying a higher HB threshold (≥87.1), SBP reduction ≥5 mmHg occurred in 50.0% (95% CI 37.7–62.3) versus 43.2% (95% CI 33.0–54.1), and SBP reduction ≥10 mmHg in 50.0% (95% CI 37.8–62.3) versus 38.3% (95% CI 28.5–49.2). Similar patterns were observed for AHI ≥34.3 compared with AHI <34.3 for SBP reduction ≥5 mmHg (53.5%, 95% CI 42.0–64.6 vs. 38.6%, 95% CI 28.0–50.3) and ≥10 mmHg (49.3%, 95% CI 38.0–60.7 vs. 37.1%, 95% CI 26.8–49.9). In a multivariable logistic regression framework, the discrimination of a prespecified model for systolic blood pressure response was evaluated with and without the addition of AHI or HB. The base model showed an AUC of 0.8219 (95% CI 0.7546–0.8891). Addition of AHI (AUC 0.8184; ΔAUC −0.0035) or HB (AUC 0.8225; ΔAUC 0.0006) did not meaningfully change model discrimination. Consistently, model fit was unchanged. The Akaike Information Criterion (AIC) was 174.5 for all models.

## 4 Discussion

This study once again confirmed that CPAP exerts only a modest blood pressure–lowering effect. Patients with more severe sleep apnea showed a greater reduction in blood pressure than those with milder disease, and a similar pattern was observed in individuals with a higher hypoxic burden compared to those with a lower burden. However, neither AHI nor, for the first time, hypoxic burden was able to predict a clinically meaningful blood pressure reduction of at least 5 mmHg.

The CPAP‐induced blood pressure reduction observed in our study is comparable to the blood pressure–lowering effects reported in previous CPAP trials. Furthermore, similar to the findings of that work, CPAP did not result in a clinically relevant reduction in diastolic blood pressure in patients with a high hypoxic burden [23]. This study also showed, in line with our previous work, that AHI is not a relevant predictor of a relevant CPAP‐induced reduction in blood pressure [13].

Previous studies have demonstrated that a greater change in hypoxic burden is associated with a decrease in blood pressure [23]. In contrast to a previous report suggesting that treatment‐related reductions in hypoxic burden may independently predict improvements in systolic blood pressure, we did not observe a significant association between ΔHB and ΔSBP after adjustment for relevant covariates (Table 3, 4, Table S2). This discrepancy may be related to differences in baseline blood pressure levels, the high prevalence of antihypertensive treatment in our cohort, or methodological differences in study design and BP assessment.

Moreover, the severity of sleep apnea—quantified by the degree of nocturnal hypoxemia—has been shown to correlate with the extent of CPAP‐induced blood pressure reduction [11, 26]. Beyond AHI and hypoxic burden, additional PSG‐derived parameters may further explain blood pressure changes and cardiovascular risk in OSA. Measures reflecting cumulative and depth‐related hypoxemia appear particularly relevant. For example, time spent with oxygen saturation below 90% (T90) has been independently associated with prevalent hypertension [27]. Similarly, oxygen desaturation index (ODI) and microarousal index (MAI) have been linked to BP elevation, suggesting that both intermittent hypoxia and sleep fragmentation may contribute to hypertension in OSA [28]. Event‐level analyses further indicate that the nadir oxygen saturation during respiratory events predicts acute nocturnal BP surges independent of event duration, supporting the concept that hypoxemia severity rather than event frequency drives acute BP responses [29].

However, the relationship between hypoxemia metrics and BP may be influenced by confounders such as obesity, as some associations lose significance after adjustment for body mass index [30]. In addition, baseline BP status appears to be a major determinant of CPAP‐related BP reduction, with meaningful BP decreases mainly observed in patients with uncontrolled hypertension [31]. Importantly, hypoxemia metrics may still capture cardiovascular risk beyond BP effects, as TST90 has been shown to independently predict major adverse cardiovascular events (MACE), whereas AHI does not [32]. These findings highlight that PSG parameters beyond AHI may provide complementary information for blood pressure response and cardiovascular risk stratification in OSA. Our results indicate that both a higher AHI and an increased hypoxic burden are associated with higher systolic blood pressure levels. Patients with an HB ≥ 57.5 %min/h showed a mean systolic BP of 136.5 mmHg, compared with 131.9 mmHg in those with an HB < 57.5 %min/h. A similar pattern was observed for AHI: individuals with an AHI ≥ 34.3/h had a mean systolic BP of 136.9 mmHg, whereas those with an AHI < 34.3/h reached 131.5 mmHg. Similarly, the absolute mean blood pressure reduction was higher in high HB group (−3.3 mmHg) and the high AHI group (−3.9 mmHg). Furthermore, based on the high‐risk OSA definition proposed by Azarbarzin et al., we conducted an analysis in patients with an HB ≥ 87.7 %min/h. This subgroup demonstrated similar reduction, exhibiting a baseline systolic blood pressure of 137.7 mmHg and a CPAP‐induced reduction of −3.4 mmHg. All data revealed no statistical significance and demonstrated only a trend. However, these findings could not be translated into a predictive value of HB or AHI for a clinically relevant blood pressure reduction of 5 mmHg in the ROC analysis. The corresponding AUC values were close to 0.5, indicating the absence of a clinically meaningful association. Likewise, the correlation analysis did not reveal a relevant relationship between the magnitude of blood pressure reduction and baseline AHI or HB. Therefore, we conclude that hypoxic burden does not represent a reliable predictor of a clinically relevant blood pressure decrease but rather appears to function as a surrogate marker for higher baseline blood pressure levels.

Our study has several limitations. We use single office blood pressure measurement in this study. This may have a limitation, so that a bias by an initial white coat effect or a habituation on readmission remain elusive. Office blood pressure was assessed using a single measurement at each visit; therefore, measurement variability cannot be excluded, which may have attenuated the observed BP response to CPAP and reduced the ability to detect associations between HB/AHI and BP changes. The influence on an additional night lowering of blood pressure also remains unclear. However, recent work has shown a correlation between systolic blood pressure in ambulatory blood pressure measurement and office measurement [33]. Noteworthy, baseline systolic BP was only 134.2 mmHg in the present study population. Thus, hypertension was frequent (66%) but well managed by pharmacological treatment prior to administration CPAP therapy.

The study is limited by its study size and its retrospective design. However, the small sample size results from the very strict inclusion and exclusion criteria, as well as the limited clinical availability and only recently established routine measurement of hypoxic burden in polysomnography. Despite its limitations, the study demonstrates notable strengths. Full polysomnography was performed, providing more accurate baseline data than polygraphy, and the cohort demonstrated a very high level of PAP usage. Moreover, the study population represented a real‐world sleep laboratory cohort, rather than secondary prevention data from patients with established cardiovascular disease. Future multicenter studies with greater sample sizes should further evaluate hypoxic burden or other PSG parameters beyond AHI as a potential predictive marker for CPAP‐induced blood pressure reduction.

## Funding

The authors have nothing to report.

## Ethics Statement

The study was conducted in accordance with the declaration of Helsinki and was approved by the ethics committee of Ruhr‐University Bochum (registration number 15–5279).

## Consent

The need for patient approval and informed consent was waived by ethics committee of Ruhr‐University Bochum due to the retrospective nature of the study.

## Conflicts of Interest

The authors have declared no conflicts of interest.

## Permission to Reproduce Material From Other Sources

No material from other sources was reproduced in this work.

## Supporting information




**Supporting Information Table 1**: Spearman Correlation Coefficients of hypoxic burden (HB) and apnea–hypopnea index (AHI) and blood pressure change.
**Supporting Information Table 2**: Multivariable linear regression was performed to evaluate the association between changes in sleep apnea severity (ΔAHI and ΔHB) and change in systolic blood pressure (ΔSBP) after CPAP therapy, adjusting for baseline systolic blood pressure, antihypertensive treatment intensity (>3 vs. ≤3 drugs), CPAP adherence, follow‐up time, BMI, age, sex, and comorbidities. Results are presented as *β*‐coefficients with 95% confidence intervals (CI). *n* = 141; adjusted *R* ^2^ = 0.40.

## Data Availability

The data that support the findings of this study are available from the corresponding author, SB, upon reasonable request.
