# Supplementary material for: Apnea–Hypopnea Index Versus Hypoxic Burden as Predictors of Blood Pressure Response to Continuous Positive Airway Pressure Treatment in Patients With Obstructive Sleep Apnoe
Source: J Clin Hypertens (Greenwich). 2026 May 22;28(5):e70262. doi: 10.1111/jch.70262 (PMC13240057; doi:10.1111/jch.70262)
Supplement: Supplementary file 1 — Supporting Information Table 1: Spearman Correlation Coefficients of hypoxic burden (HB) and apnea–hypopnea index (AHI) and blood pressure change. Supporting Information Table 2: Multivariable linear regression was performed to evaluate the association between changes in sleep apnea severity (ΔAHI and ΔHB) and change in systolic blood pressure (ΔSBP) after CPAP therapy, adjusting for baseline systolic blood pressure, antihypertensive treatment intensity (>3 vs. ≤3 drugs), CPAP adherence, follow‐up time, BMI, age, sex, and comorbidities. Results are presented as β‐coefficients with 95% confidence intervals (CI). n = 141; adjusted R 2 = 0.40. [file JCH-28-e70262-s001.docx]

**Supplemental Table 1**: Spearman Correlation Coefficients of hypoxic burden (HB) and apnea–hypopnea index (AHI) and blood pressure change

|  | Spearman r | P value |
| --- | --- | --- |
| HB vs. RR change (sys) | -0.13 | 0.1269 |
| HB vs. RR change (dia) | -0.03 | 0.7607 |
|  |  |  |
| AHI vs. RR change (sys) | -0.15 | 0.0667 |
| AHI vs. RR change (dia) | -0.04 | 0.6797 |

**Supplemental Table 2.** Multivariable linear regression was performed to evaluate the association between changes in sleep apnea severity (ΔAHI and ΔHB) and change in systolic blood pressure (ΔSBP) after CPAP therapy, adjusting for baseline systolic blood pressure, antihypertensive treatment intensity (>3 vs ≤3 drugs), CPAP adherence, follow-up time, BMI, age, sex, and comorbidities. Results are presented as β-coefficients with 95% confidence intervals (CI). n = 141; adjusted R² = 0.40.

| *Variable* | *ß-Coefficient* | *95%CI* | *P* |
| --- | --- | --- | --- |
| *Apnea–Hypopnea Index change (*ΔAHI*)* | -0.06 | -0.25 to 0.13 | 0.5236 |
| *Hypoxic Burden change (*Δ*HB)* | 0.01 | -0.03 to 0.04 | 0.7094 |
| *Baseline systolic BP(mmHg)* | -0.73 | -0.88 to -0.57 | <0.0001 |
| *Antihypertensive regimen (>3 vs ≤3 drugs)* | -2.5 | -11 to 6.2 | 0.5706 |
| *CPAP usage (hours/night)* | 0.61 | -0.44 to 1.7 | 0.2524 |
| *Follow up time (days)* | 0.01 | -0.03 to 0.05 | 0.6860 |
| *Body mass index (kg/m²)* | 0.56 | 0.17 to 0.94 | 0.0054 |
| *Age (years)* | 0.12 | -0.08 to 0.33 | 0.2415 |
| *Sex (male vs female)* | -4.7 | -10 to 0.66 | 0.0852 |
| *Arterial hypertension* | 1.2 | -4.0 to 6.5 | 0.6433 |
| *Diabetes* | -3.4 | -9.4 to 2.7 | 0.2697 |
| *Coronary heart disease* | -0.49 | -7.4 to 6.4 | 0.8871 |
| *Hyperlipidemia* | 2.1 | -3.1 to 7.3 | 0.4197 |
